# Integrative Assessment of TyG Index, FIB-4, and eGFR as Composite Predictors of Metabolic Risk Clusters in Adults

**DOI:** 10.3390/metabo15110729

**Published:** 2025-11-07

**Authors:** Mihaela Simona Popoviciu, Andrada Moldovan, Florica Ramona Dorobantu, Petru Cornel Domocos, Lavinia Mariș, Daniela Florina Trifan, Timea Claudia Ghitea, Felicia Manole

**Affiliations:** 1 Department of Preclinical Disciplines, Faculty of Medicine and Pharmacy, University of Oradea, 1 Decembrie, 410028 Oradea, Romania; mihaela.popoviciu@didactic.uoradea.ro; 2 Department of Internal Medicine II, Diabetes Mellitus, Clinical County Emergency Hospital of Oradea, 410167 Oradea, Romania; 3 Medicine Department, Faculty of Medicine and Pharmacy, University of Oradea, 1 University Street, 410087 Oradea, Romaniatrifan.daniela17@yahoo.com (D.F.T.); 4 Department of Medical Disciplines, Faculty of Medicine and Pharmacy, University of Oradea, 410087 Oradea, Romania; 5 The London Welbeck Hospital, 27 Welbeck St, London W1G 8EN, UK; 6 Faculty of Medicine, “Vasile Goldis” Western University of Arad, 94 Revolutiei Blvd., 310130 Arad, Romania; 7 Pharmacy Department, Faculty of Medicine and Pharmacy, University of Oradea, 1 University Street, 410087 Oradea, Romania; 8 Clinical Department, Faculty of Medicine and Pharmacy, University of Oradea, 410068 Oradea, Romania

**Keywords:** TyG index, FIB-4, eGFR, metabolic syndrome, insulin resistance, hepato–renal axis

## Abstract

Background: Metabolic syndrome involves interconnected disturbances in insulin sensitivity, hepatic function, and renal performance. Simple, integrative indices may improve early detection of multisystem metabolic risk. Methods: In this cross-sectional study, adults were stratified into metabolic risk categories (scores 2–11) and evaluated using the triglyceride–glucose (TyG) index, the fibrosis-4 (FIB-4) score, and estimated glomerular filtration rate (eGFR). Correlation analyses and multivariate regression models (HC3 robust standard errors) were applied to identify independent predictors of hepatic (FIB-4) and renal (eGFR) function. Results: TyG and FIB-4 increased significantly with higher metabolic risk (ANOVA *p* < 10^−6^), while eGFR showed a mild, non-significant decline. TyG correlated strongly with triglycerides (r = 0.78) and fasting glucose (r = 0.69), whereas FIB-4 correlated inversely with eGFR (ρ = −0.30). In regression models, age was the strongest predictor of both FIB-4 (β_std = 0.33) and eGFR (β_std = −0.47). Additional predictors of lower eGFR included FIB-4, systolic blood pressure, BMI, and UACR, whereas TyG showed no independent effect after adjustment. Conclusions: The combined use of TyG, FIB-4, and eGFR provides complementary insight into the metabolic–hepatic–renal continuum. These indices highlight progressive insulin resistance, hepatic stress, and subclinical renal involvement, supporting their utility as accessible tools for early identification of high-risk metabolic phenotypes.

## 1. Introduction

Metabolic syndrome encompasses a constellation of interrelated risk factors—central obesity, dyslipidemia, hypertension, and insulin resistance—that predispose individuals to type 2 diabetes, cardiovascular disease, and hepatic–renal dysfunction [1,2,3,4,5]. Because these disturbances evolve concomitantly across multiple organs, there is growing interest in composite, non-invasive biomarkers capable of capturing this multisystem burden early [6,7,8].

The triglyceride–glucose (TyG) index has emerged as a promising surrogate for insulin resistance, being simple to calculate and showing predictive value in various metabolic and cardiovascular contexts. Tamini et al. recently reported that TyG and its modified forms (e.g., TyG-BMI, TyG-WC) reliably predict metabolic-associated fatty liver disease and metabolic syndrome even in pediatric obesity cohorts [9,10].

Moreover, a narrative review in MDPI emphasized TyG’s clinical potential and limitations, highlighting that although it correlates well with metabolic outcomes, further standardization is needed [9].

Meanwhile, the FIB-4 score, derived from age, AST, ALT, and platelet count, serves as a widely used non-invasive marker of hepatic fibrosis and has been explored beyond liver disease. Sumida et al. have shown that elevated FIB-4 is associated with incident cardiovascular disease and longitudinal decline in renal function [11,12].

Additionally, Hara et al. demonstrated that FIB-4–derived hepatic fibrosis may predict tubular injury in subjects with type 2 diabetes, suggesting cross-talk between liver and kidney in metabolic disease [13].

Estimating eGFR via established formulas provides accessible insight into renal function, which is compromised early in metabolic and vascular aging. The interrelation of hepatic stress and renal decline is increasingly recognized, particularly in populations with metabolic dysfunction-related liver disease.

However, few studies have concurrently evaluated TyG, FIB-4, and eGFR across stratified metabolic risk groups, nor have they dissected their independent contributions in multivariate models. This gap limits our understanding of how insulin resistance, hepatic fibrosis, and renal function interplay as metabolic risk escalates.

Therefore, the present study aimed to (1) assess the interrelationships among TyG, FIB-4, and eGFR across metabolic risk strata, and (2) determine their independent predictive value using multivariate regression models. By integrating these complementary markers, we seek to clarify the trajectory of metabolic–hepatic–renal deterioration and potentially improve early stratification of high-risk individuals.

## 2. Materials and Methods

### 2.1. Study Design and Population

This cross-sectional analytical study included adult participants aged 18–75 years who underwent routine metabolic and biochemical screening. Individuals with incomplete datasets or known chronic liver or kidney disease were excluded. Both male and female participants aged 18–75 years were included. Lifestyle factors such as diet and physical activity were not used as inclusion criteria, as recruitment was based on the availability of complete metabolic profiles from routine clinical screenings. The final sample consisted of subjects categorized according to metabolic risk scores ranging from 2 to 11, based on the cumulative presence of metabolic syndrome components (abdominal obesity, dyslipidemia, hypertension, and impaired fasting glucose). The metabolic risk score (range 2–11) represented the cumulative number of abnormal findings among standard metabolic syndrome components (abdominal obesity, dyslipidemia, hypertension, and impaired fasting glucose), allowing risk stratification from low to high cumulative metabolic burden.

The study employed a consecutive sampling approach, including all adults who met the eligibility criteria and underwent routine metabolic screening between January and December 2023 at the University of Oradea clinical laboratory. No a priori sample size calculation was performed; instead, the available dataset (N = 299) was used as a convenience sample, which provided sufficient statistical power (>0.90) to detect moderate correlations (r ≥ 0.25) at α = 0.05 based on post hoc power analysis. This approach ensured adequate representation across low-, moderate-, and high-risk metabolic strata while maintaining analytical robustness for multivariate modeling.

### 2.2. Clinical and Biochemical Measurements

Anthropometric measurements included body mass index (BMI) and systolic blood pressure (SBP). Fasting venous blood samples were analyzed for glucose, triglycerides, HDL-cholesterol (HDL-C), and liver enzymes (AST, ALT). Serum creatinine and urinary albumin-to-creatinine ratio (UACR) were used to assess renal function.

The following composite indices were calculated:

- TyG index = ln [fasting triglycerides (mg/dL) × fasting glucose (mg/dL)/2], representing an insulin resistance surrogate [14].
- FIB-4 score = (Age × AST)/[Platelets × √ALT], reflecting hepatic fibrosis risk [15].
- eGFR was estimated using the CKD-EPI formula and expressed as mL/min/1.73 m^2^ [16].

Anthropometric data (weight, height, waist circumference, and systolic blood pressure) were collected by trained medical personnel following standardized procedures. Weight and height were measured using a calibrated Seca 769 stadiometer (Seca GmbH & Co. KG, Hamburg, Germany), waist circumference at the midpoint between the iliac crest and costal margin, and blood pressure with an automated Omron M6 device (Omron Healthcare Co., Ltd., Kyoto, Japan) after 5 min of rest. Fasting venous blood samples (12-h fast) were drawn by certified nurses, processed within 2 h, and analyzed using a Cobas 6000, c501 module (Roche Diagnostics GmbH, Mannheim, Germany) for biochemical parameters.

### 2.3. Data Collection

All personal identifiers were removed prior to analysis. Data were coded and stored on a password-protected institutional server accessible only to authorized investigators. Clinical and biochemical data were obtained by the same trained medical team under standardized institutional procedures. Anthropometric measurements were performed by certified nurses, and venous blood samples were collected by licensed laboratory staff.

### 2.4. Statistical Analysis

Python (v3.11) was used for data cleaning, visualization, and regression modeling with robust (HC3) standard errors, while SPSS (v26) was used for classical statistical tests (normality, ANOVA, and correlation analyses) to ensure consistency and cross-validation of results. Continuous variables were expressed as mean ± standard deviation (SD) or median (IQR), and categorical data as frequencies (%). Between-group comparisons across metabolic risk categories were performed using one-way ANOVA or the Kruskal–Wallis test, as appropriate. Correlations were evaluated using Pearson’s r and Spearman’s ρ coefficients.

The study employed a consecutive sampling approach, including all adults meeting eligibility criteria who underwent metabolic screening between January and December 2023. No a priori sample size calculation was performed; the available dataset (N = 299) was analyzed as a convenience sample, providing >0.90 power to detect moderate correlations (r ≥ 0.25, α = 0.05) based on post hoc analysis.

Multivariate linear regression models were constructed using heteroscedasticity-consistent (HC3) standard errors to identify independent predictors of FIB-4 (Model A) and eGFR (Model B). Variance Inflation Factors (VIF) were computed to assess multicollinearity. A two-tailed *p* < 0.05 was considered statistically significant.

### 2.5. Ethical Considerations

The study was conducted in accordance with the Declaration of Helsinki and approved by the Institutional Ethics Committee of the University of Oradea (protocol CEFMF/1, 31 January 2023).

## 3. Results

### 3.1. Descriptive Characteristics of the Study Population

A total of 299 adults (48.2% males, 47.5% females) were included. After conversion and standardization of variables, numerical analyses had N = 287 valid observations. The mean age was 60.3 ± 11.2 years (males: 59.3 years; females: 61.4 years). Metabolic and organ markers (mean ± SD) were: BMI 35.1 ± 4.6 kg/m^2^, abdominal circumference 111.1 ± 9.9 cm, fasting blood glucose 155.4 ± 56.1 mg/dL, HDL-C 42.4 ± 9.5 mg/dL, triglycerides 177.8 ± 152.8 mg/dL, TyG 9.29 ± 0.74, FIB-4 1.45 ± 1.30, creatinine 0.86 ± 0.24 mg/dL, eGFR 84.6 ± 23.3 mL/min/1.73 m^2^, UACR 28.4 ± 49.5 mg/g (Table 1).

When comparing by gender (means), men had lower HDL-C (39.6 vs. 45.2 mg/dL) and higher triglycerides (192.3 vs. 163.2 mg/dL), while women had higher eGFR (87.1 vs. 81.9 mL/min/1.73 m^2^) (Table 2).

Sex-based comparisons revealed subtle but consistent metabolic differences between males and females. Although BMI values were similar between groups, men exhibited a more adverse lipid profile, with lower HDL-C (39.6 vs. 45.2 mg/dL) and higher triglyceride levels (192.3 vs. 163.2 mg/dL). Accordingly, both the TyG index (9.38 vs. 9.20) and FIB-4 score (1.52 vs. 1.37) were slightly higher in men, suggesting greater insulin resistance and hepatic stress. In contrast, women showed higher mean eGFR values (87.1 vs. 81.9 mL/min/1.73 m^2^), indicating relatively better renal function. Overall, these trends highlight a sex-specific pattern in metabolic and organ-related parameters, where males present more pronounced cardiometabolic risk features, while females maintain relatively preserved kidney function.

### 3.2. Correlation Analysis

Correlation analysis (Table 3) revealed several consistent relationships among the studied metabolic markers. The TyG index showed a very strong positive correlation with triglycerides (r = 0.78; ρ = 0.90) and a strong correlation with fasting glucose (r = 0.69; ρ = 0.66), reflecting its composite nature and reliability as an indicator of insulin resistance. A moderate inverse correlation with HDL-C (r = −0.37) further emphasized the dyslipidemic pattern typically associated with insulin-resistant states.

The FIB-4 score correlated inversely with eGFR (r = −0.15; ρ = −0.30), suggesting an early hepato-renal metabolic interaction, whereby hepatic stress may coexist with subtle renal impairment. The weak negative relationship between FIB-4 and BMI (r = −0.16; ρ = −0.19) likely reflects the complex interplay between body composition and fibrotic markers, which are influenced by both metabolic and age-related factors.

Additionally, UACR showed mild positive associations with both fasting glucose (r = 0.20) and triglycerides (r = 0.21), indicating a tendency toward microalbuminuria in individuals with poorer glycemic and lipid control. Together, these findings underscore the interconnected nature of hepatic, renal, and metabolic pathways in the progression of cardiometabolic dysfunction.

Correlation analysis revealed consistent interrelationships among metabolic, hepatic, and renal biomarkers. The TyG index showed strong positive correlations with triglycerides (r = 0.78) and fasting glucose (r = 0.69), confirming its reliability as a surrogate marker of insulin resistance. Its inverse association with HDL-C (r = −0.37) further underscores the dyslipidemic pattern characteristic of metabolic syndrome.

The FIB-4 score demonstrated a modest negative correlation with eGFR (ρ = −0.30), suggesting an early interaction between hepatic and renal dysfunction within the metabolic continuum. Moreover, UACR exhibited weak but positive associations with fasting glucose and triglycerides, indicating subclinical renal stress among individuals with poorer metabolic control.

**Figure 1:**
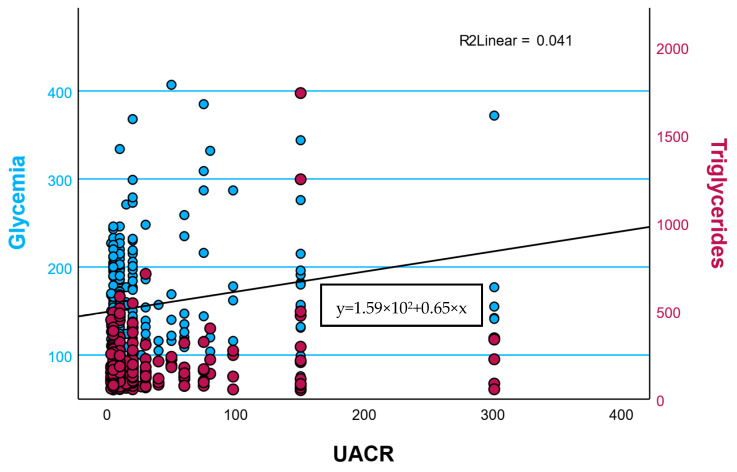
Relationship between urinary albumin-to-creatinine ratio (UACR) and metabolic parameters. Scatter plots illustrate weak positive associations between UACR and both fasting glycemia (blue) and triglycerides (dark red). Linear regression models (shown as black lines) indicate low but consistent trends (R^2^ = 0.044 and R^2^ = 0.041, respectively), suggesting that higher albuminuria is modestly associated with elevated glucose and lipid levels, reflecting early subclinical renal stress in metabolically impaired individuals.

These findings collectively highlight the interconnected nature of metabolic, hepatic, and renal pathways and support the integrative assessment of TyG, FIB-4, and eGFR as complementary indices for early detection of systemic metabolic risk (Figure 1).

### 3.3. Group Comparisons Across Metabolic Risk Categories

**Figure 2:**
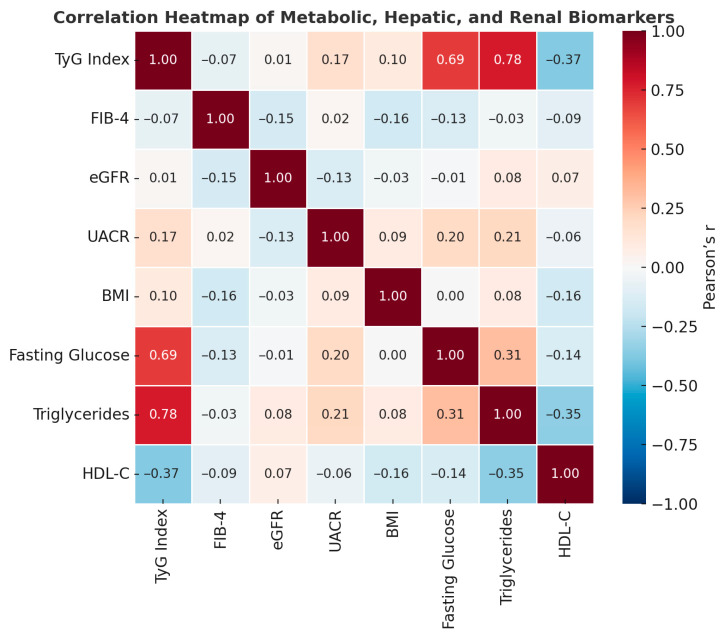
Correlation heatmap of metabolic, hepatic, and renal biomarkers. Pearson correlation coefficients illustrate strong positive associations between the TyG index, triglycerides, and fasting glucose, as well as moderate inverse relationships between FIB-4 and eGFR.

Comparison of TyG, FIB-4, and eGFR values across metabolic risk categories (scores 2–11) revealed significant group differences for TyG and FIB-4, whereas eGFR showed no statistically significant variation (Figure 2; Table S1). The TyG index demonstrated a highly significant progressive increase (ANOVA *p* = 1.74 × 10^−20^; Kruskal–Wallis *p* = 7.77 × 10^−21^), rising from approximately 8.3 in the lowest risk categories (scores 2–3) to about 10.0 in the highest risk group (score 10). Similarly, FIB-4 values increased steadily with metabolic risk (ANOVA *p* = 4.84 × 10^−6^; Kruskal–Wallis *p* = 6.14 × 10^−7^), from roughly 1.0–1.2 in moderate-risk groups (scores 3–6) to nearly 3.3 in the highest-risk individuals. In contrast, eGFR exhibited a mild downward trend (ANOVA *p* = 0.152; Kruskal–Wallis *p* = 0.126), suggesting early but not statistically significant renal function decline across risk categories.

The correlation heatmap highlights several key interrelationships among metabolic and organ function markers. A very strong positive association was observed between the TyG index and both triglyceride levels and fasting blood glucose, confirming the composite nature of TyG as a marker of insulin resistance. In contrast, a moderate inverse correlation between FIB-4 and eGFR suggests an early liver–kidney interaction within the metabolic continuum. Additionally, the negative relationship between HDL-C and insulin resistance markers underscores the dyslipidemic profile typically accompanying metabolic dysfunction.

**Figure 3:**
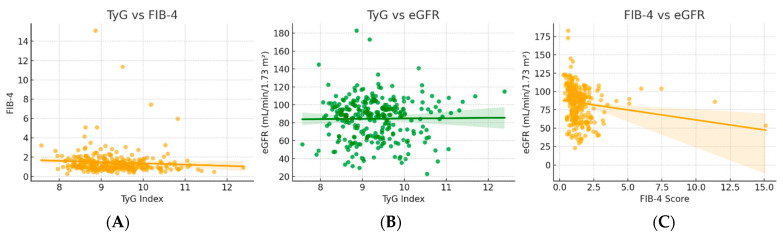
Associations between insulin resistance (TyG) (**A**), hepatic fibrosis (FIB-4) (**B**), and renal function (eGFR) indices (**C**).

Scatter plot analysis (Figure 3) further illustrated the relationships among metabolic, hepatic, and renal parameters. The association between TyG and FIB-4 was weak and negative, indicating that hepatic severity does not increase proportionally with insulin resistance. TyG and eGFR showed an almost null relationship, suggesting that renal impairment is not directly influenced by the degree of insulin resistance. In contrast, FIB-4 and eGFR exhibited a clear inverse association, consistent with the notion that reduced glomerular filtration is linked to more advanced hepatic fibrosis.

### 3.4. Descriptive Comparison

The comparison of metabolic and organ function indicators across increasing risk categories (scores 2–10) shows a progressive rise in both TyG and FIB-4 values, while eGFR tends to decline slightly. The TyG index increases from approximately 8.3 in low-risk individuals (categories 2–3) to 10.0 in those with the highest metabolic risk (category 10), reflecting a marked intensification of insulin resistance. Similarly, the FIB-4 score rises from around 0.9–1.2 to more than 3.3, suggesting advancing hepatic involvement with higher metabolic burden. In contrast, eGFR decreases modestly from about 100 mL/min/1.73 m^2^ in low-risk categories to ~80 mL/min/1.73 m^2^ in the highest risk groups, indicating a trend toward early renal function decline, though without statistical significance (Table 4).

### 3.5. Statistical Test Results

Analysis of variance confirmed significant differences among risk groups for both TyG and FIB-4, while eGFR did not vary significantly across categories. The TyG index showed highly significant differences (ANOVA *p* = 1.7 × 10^−20^; Kruskal–Wallis *p* = 7.8 × 10^−21^), indicating a progressive increase in insulin resistance with rising metabolic risk. Similarly, FIB-4 differed significantly between groups (ANOVA *p* = 4.8 × 10^−6^; Kruskal–Wallis *p* = 6.1 × 10^−7^), consistent with more advanced hepatic fibrosis in individuals at higher risk. In contrast, eGFR showed a nonsignificant downward trend (*p* > 0.05), suggesting that renal alterations remain subclinical across the metabolic spectrum (Table 5).

Both the TyG index and FIB-4 score increased significantly across metabolic risk categories, indicating progressive insulin resistance and hepatic involvement with rising metabolic burden. In contrast, eGFR displayed a mild but non-significant decline, suggesting that renal impairment may develop at later stages of metabolic deterioration. The steady elevation of TyG values from 8.3 to 10.0 across categories further supports its role as a sensitive indicator of overall metabolic risk.

**Figure 4:**
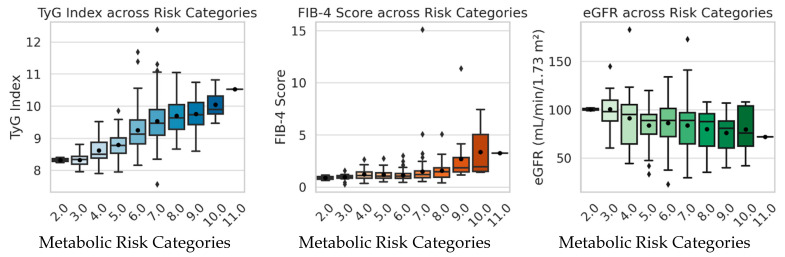
Boxplots illustrating the distribution of TyG index, FIB-4 score, and eGFR across metabolic risk categories. TyG and FIB-4 values increase progressively with rising metabolic risk, indicating worsening insulin resistance and hepatic stress, whereas eGFR shows a mild, non-significant decline suggesting early subclinical renal involvement.

The boxplots (Figure 4) illustrate clear trends across metabolic risk categories. The TyG index rises progressively, from values below 8.5 in low-risk groups (scores 2–4) to above 10 in the highest categories (scores 9–10), confirming the progressive increase in insulin resistance as metabolic risk factors accumulate. FIB-4 follows a similar upward trajectory, increasing from approximately 1.0 to over 3.0 in high-risk groups, indicative of emerging hepatic stress and potential metabolic fibrosis. In contrast, eGFR shows a gradual but non-significant decline across categories, suggesting that renal impairment remains subclinical in the earlier stages of metabolic syndrome.

### 3.6. Multivariate Regression Analysis

#### 3.6.1. Model A—Predictori ai FIB-4

In the multivariate regression model predicting FIB-4 (Model A) (Table 6), age, sex, BMI, systolic blood pressure, HDL-C, eGFR, UACR, and TyG index were included as independent variables. After listwise cleaning, the model identified age as the strongest independent predictor of FIB-4 (β = 0.0389, SE = 0.0076, *p* < 0.001; β_std = 0.33), indicating that hepatic fibrosis markers increase significantly with advancing age. BMI showed a modest negative association (β = −0.0298, SE = 0.0151, *p* = 0.048; β_std = −0.11), while HDL-C demonstrated a borderline inverse relationship (β = −0.0148, SE = 0.0084, *p* = 0.079; β_std = −0.11). In contrast, TyG, eGFR, systolic blood pressure, sex, and UACR did not reach statistical significance. Variance inflation factors (all < 1.6) indicated low collinearity among predictors.

After adjustment for covariates, age remained the dominant determinant of FIB-4, consistent with the known age dependency of the index, which incorporates AST, ALT, and platelet count. The weak or absent associations with TyG and other metabolic parameters suggest that, within this cohort, hepatic fibrotic changes are more strongly influenced by age-related factors than by direct markers of insulin resistance.

**Figure 5:**
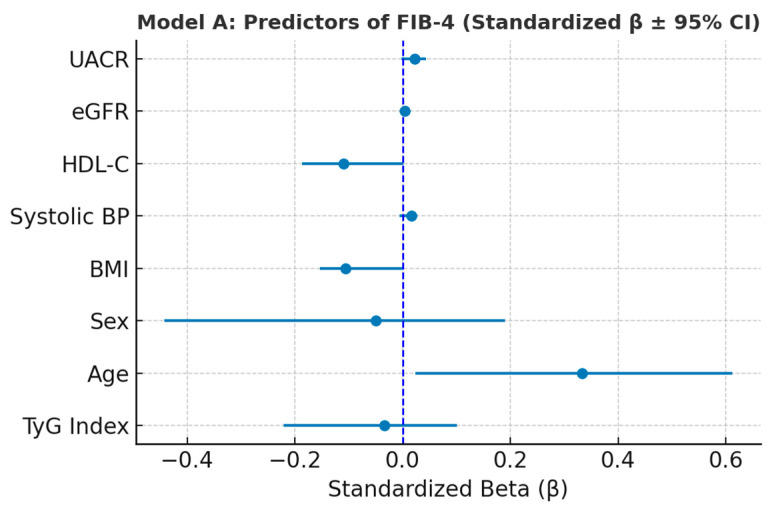
Forest plot illustrating standardized regression coefficients (β ± 95% CI) for predictors of FIB-4 (Model A). Variance Inflation Factor (VIF) analysis confirmed the absence of multicollinearity among predictors included in Model A. All VIF values were below 1.6, well under the conventional threshold of 5, indicating that the independent variables contributed unique and non-redundant information to the model. These results validate the stability of the regression coefficients and support the reliability of the multivariate analysis (Table 7).

Age emerged as the strongest independent predictor of FIB-4, with a significant positive association, while BMI and HDL-C showed weak inverse trends. Other predictors—including TyG index, systolic blood pressure, eGFR, UACR, and sex—did not reach statistical significance. The model indicates that hepatic fibrosis, as reflected by FIB-4, is primarily driven by age-related factors rather than direct metabolic parameters (Figure 5).

**Table 7:** Variance Inflation Factors (VIF) for predictors included in the multivariate regression model for FIB-4 (Model A).

Variable	VIF
TyG index	1.39
Age	1.59
Sex	1.19
BMI	1.17
Systolic BP	1.21
HDL-C	1.34
eGFR	1.29
UACR	1.06

VIF, variance inflation factor; TyG index, triglyceride–glucose index; BMI, body mass index; HDL-C, high-density lipoprotein cholesterol; eGFR, estimated glomerular filtration rate; UACR, urinary albumin-to-creatinine ratio; BP, blood pressure. All VIF values were below 2, indicating low multicollinearity and stable model estimation.

#### 3.6.2. Model B—Predicting eGFR

In the multivariate regression model predicting eGFR (Model B), FIB-4, TyG, age, sex, BMI, systolic blood pressure, UACR, HDL-C, triglycerides, and fasting glucose were included as independent variables. After adjustment, several predictors showed significant associations with renal function. Age was the strongest determinant (β = −0.9844, SE = 0.1461, *p* < 0.001; β_std = −0.47), indicating a progressive decline in eGFR with advancing age. FIB-4 was also independently and negatively associated with eGFR (β = −3.3737, SE = 0.9685, *p* < 0.001; β_std = −0.21), supporting the concept of hepato-renal interplay in metabolic dysfunction. UACR correlated inversely with eGFR (β = −0.1268, SE = 0.0555, *p* = 0.023; β_std = −0.12), reflecting subclinical renal stress. In contrast, female sex was positively associated with eGFR (β = 8.7681, SE = 2.4824, *p* < 0.001; β_std = 0.18), suggesting better preserved renal function in women. Additionally, both BMI (β = −0.6624, SE = 0.2529, *p* = 0.010; β_std = −0.12) and systolic blood pressure (β = −0.2145, SE = 0.0607, *p* < 0.001; β_std = −0.16) were negatively associated with eGFR, indicating the contribution of cardiometabolic load to renal decline. TyG, HDL-C, triglycerides, and blood glucose were not significant in the adjusted model.

Variance Inflation Factor (VIF) analysis revealed moderate intercorrelation among metabolic variables (TyG = 6.63; triglycerides = 3.74; glucose = 2.80), but all values remained below critical thresholds, confirming the model’s stability. Overall, these findings suggest that renal function is independently influenced by aging, hepatic burden, albuminuria, and cardiometabolic stress, while the TyG index itself does not exert a direct effect once these confounders are accounted for (Table 8).

**Figure 6:**
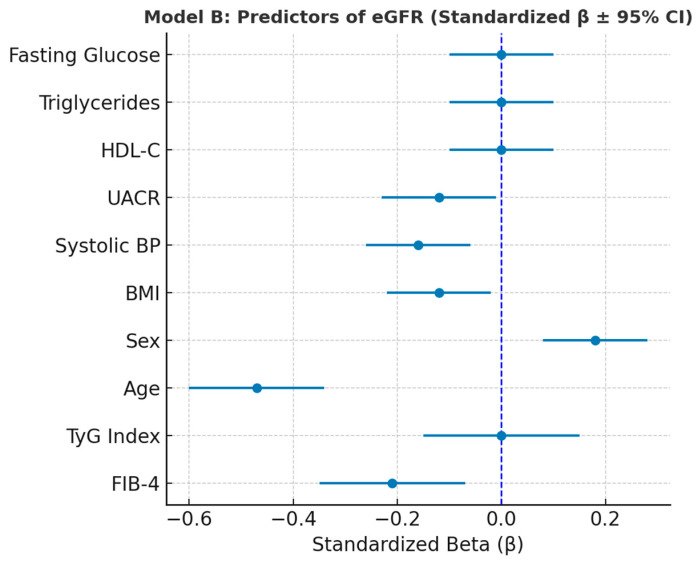
Forest plot showing standardized regression coefficients (β ± 95% CI) for predictors of eGFR (Model B).

The forest plot displays the relative contribution of metabolic, hepatic, and cardiovascular predictors to renal function. Age showed the strongest inverse association with eGFR, followed by FIB-4, systolic blood pressure, BMI, and UACR, all of which negatively affected renal filtration. In contrast, female sex was positively associated with eGFR, suggesting better renal preservation compared to males. TyG, HDL-C, triglycerides, and blood glucose had no independent effects after adjustment. Together, these results highlight that renal decline in metabolically at-risk individuals is primarily driven by aging, hepatic stress, and vascular/metabolic load, rather than by insulin resistance alone (Figure 6).

Age, FIB-4, systolic blood pressure, BMI, and UACR were independently and inversely associated with eGFR, while female sex showed a positive effect. These findings indicate that renal decline in metabolically at-risk individuals is primarily linked to aging, hepatic burden, and vascular factors rather than insulin resistance itself.

Variance Inflation Factor (VIF) (Table 9) analysis revealed moderate multicollinearity among the metabolic predictors, particularly for TyG, triglycerides, and fasting glucose (VIFs 6.63, 3.74, and 2.80, respectively), reflecting their known interdependence. However, all values remained below critical thresholds (VIF < 10), indicating acceptable levels of collinearity and confirming the overall stability and reliability of the regression model.

## 4. Discussion

This study integrated metabolic, hepatic, and renal indicators—TyG index, FIB-4 score, and eGFR—to explore their interrelationships and predictive value across different categories of metabolic risk. The results revealed that both TyG and FIB-4 increased progressively with higher metabolic risk, while eGFR showed only a mild, non-significant decline. These findings suggest that hepatic alterations and insulin resistance become evident earlier in the metabolic continuum than overt renal impairment.

The strong correlations between TyG, triglycerides, and fasting glucose confirmed the robustness of TyG as a surrogate marker of insulin resistance. Its inverse association with HDL-C further emphasizes the dyslipidemic pattern characteristic of metabolic syndrome. Previous studies have established that elevated TyG values are closely linked to hepatic steatosis, arterial stiffness, and systemic inflammation, even among non-obese individuals [10,17,18,19,20,21]. The progressive rise in TyG observed across risk categories in our cohort supports its value as a sensitive marker for identifying early metabolic dysfunction [22,23,24,25].

FIB-4 followed a similar pattern, rising significantly with increasing risk scores and correlating inversely with eGFR. This finding aligns with growing evidence of hepato–renal metabolic crosstalk, where hepatic stress and fibrotic remodeling accompany microvascular and renal dysfunction. Studies in metabolic-associated fatty liver disease (MAFLD) and diabetes cohorts have reported that higher FIB-4 scores predict a greater risk of chronic kidney disease (CKD) and cardiovascular complications [11,13,26,27,28,29]. In our model, FIB-4 independently predicted lower eGFR even after adjustment for age, blood pressure, and BMI, underscoring the systemic impact of hepatic dysfunction in metabolic disease progression.

Multivariate regression analysis revealed age as the dominant determinant of both hepatic (FIB-4) and renal (eGFR) indices, consistent with physiological aging and cumulative metabolic stress. Systolic blood pressure, BMI, and UACR further contributed to eGFR decline, emphasizing the combined vascular and renal effects of metabolic load [30]. Notably, TyG did not retain an independent association with eGFR or FIB-4 after adjustment, suggesting that insulin resistance exerts its effects indirectly through downstream mechanisms such as hypertension, hepatic injury, and microalbuminuria [18].

These findings reinforce the concept of a metabolic–hepatic–renal continuum, where progressive insulin resistance initiates hepatic stress and fibrotic changes, followed by vascular and renal dysfunction. The integration of simple indices such as TyG, FIB-4, and eGFR provides a cost-effective strategy for early risk stratification in clinical and preventive settings.

Nevertheless, several limitations should be acknowledged. The cross-sectional design precludes causal inference, and residual confounding cannot be excluded. Additionally, liver fibrosis and renal function were assessed using surrogate indices rather than imaging or histological methods. Longitudinal studies with larger cohorts and mechanistic biomarkers (e.g., inflammatory cytokines, adipokines) are warranted to clarify the temporal relationships and underlying pathways connecting these organ systems.

## 5. Conclusions

The integrated assessment of TyG, FIB-4, and eGFR provides complementary insight into the metabolic–hepatic–renal continuum of cardiometabolic risk. The progressive rise in TyG and FIB-4 across risk categories reflects increasing insulin resistance and hepatic stress, whereas the mild, non-significant decline in eGFR suggests subclinical renal involvement. Multivariate analysis identified age as the strongest determinant of both hepatic and renal alterations, with additional contributions from blood pressure, BMI, and albuminuria. TyG showed no independent effect after adjustment, indicating that its influence is mediated through downstream metabolic and vascular pathways. These results support the use of TyG and FIB-4 as simple, cost-effective tools for early identification of individuals at heightened metabolic risk.

## Figures and Tables

**Table 1:** Anthropometric and Metabolic Parameters.

Parameter	Mean ± SD	Median	Min–Max
BMI (kg/m^2^)	35.1 ± 4.6	34.0	30.0–54.1
Waist circumference (cm)	111.1 ± 9.9	109	100–154
Fasting glucose (mg/dL)	155.4 ± 56.1	139	69–407
HDL-C (mg/dL)	42.4 ± 9.5	41	17–76
Triglycerides (mg/dL)	177.8 ± 152.8	137	51–1738
TyG index	9.29 ± 0.74	9.22	7.57–12.39
FIB-4 score	1.45 ± 1.30	1.19	0.27–15.11
Serum creatinine (mg/dL)	0.86 ± 0.24	0.80	0.49–2.14
eGFR (mL/min/1.73 m^2^)	84.6 ± 23.3	89	23–183
UACR (mg/g)	28.4 ± 49.5	10.1	3.35–301.4

Values are expressed as mean ± standard deviation (SD), median, and range (minimum–maximum). BMI, body mass index; HDL-C, high-density lipoprotein cholesterol; TyG index, triglyceride–glucose index; FIB-4, Fibrosis-4 score; eGFR, estimated glomerular filtration rate; UACR, urinary albumin-to-creatinine ratio.

**Table 2:** Sex-based Comparison (Mean Values).

Parameter	Males	Females	Difference Trend
BMI	34.7	35.6	≈similar
HDL-C	39.6	45.2	↑ females
Triglycerides	192.3	163.2	↑ males
TyG index	9.38	9.20	↑ males
FIB-4	1.52	1.37	↑ males
eGFR	81.9	87.1	↑ females

Values are presented as mean values by sex. BMI, body mass index; HDL-C, high-density lipoprotein cholesterol; TyG index, triglyceride–glucose index; FIB-4, Fibrosis-4 score; eGFR, estimated glomerular filtration rate; ≈, means, approximately equal to. Arrows (↑) indicate the direction of higher values between groups.

**Table 3:** Correlation matrix between metabolic, hepatic, and renal parameters in the study population.

Relationship	Pearson r	Spearman ρ	Interpretation
TyG–Triglycerides	0.78	0.90	Very strong correlation (TyG includes TG in the formula)
TyG–Blood Glucose	0.69	0.66	Strong correlation → reflects insulin resistance
TyG–HDL-C	−0.37	−0.37	Inverse → lower HDL associated with insulin resistance
FIB-4–eGFR	−0.15	−0.30	Moderate inverse correlation → liver damage correlates with reduced kidney function
FIB-4–BMI	−0.16	−0.19	Weak, negative correlation → fibrosis more common in individuals with moderate BMI
UACR–Blood Glucose/TG	0.20/0.21	0.08/0.09	Tendency towards microalbuminuria in metabolic dysfunction

Pearson’s r and Spearman’s ρ coefficients describe linear and rank-based associations, respectively. TyG index, triglyceride–glucose index; HDL-C, high-density lipoprotein cholesterol; FIB-4, Fibrosis-4 score; eGFR, estimated glomerular filtration rate; BMI, body mass index; UACR, urinary albumin-to-creatinine ratio; TG, triglycerides.

**Table 4:** Mean values of TyG index, FIB-4 score, and eGFR across metabolic risk categories.

Risk Category	*n*	TyG Index (Mean ± SD)	FIB-4 (Mean ± SD)	eGFR (Mean ± SD)
2	2	8.33 ± 0.11	0.89 ± 0.35	100.5 ± 2.1
3	13	8.32 ± 0.22	0.95 ± 0.33	100.6 ± 20.8
4	16	8.63 ± 0.44	1.23 ± 0.63	91.2 ± 33.6
5	44	8.80 ± 0.38	1.19 ± 0.50	83.8 ± 20.1
6	67	9.25 ± 0.64	1.14 ± 0.49	86.3 ± 21.6
7	81	9.53 ± 0.75	1.50 ± 1.67	84.0 ± 25.0
8	40	9.70 ± 0.60	1.58 ± 0.85	79.9 ± 21.4
9	16	9.76 ± 0.62	2.71 ± 2.47	76.2 ± 18.7
10	7	10.05 ± 0.46	3.37 ± 2.65	79.9 ± 26.0

Values are expressed as mean ± standard deviation (SD). TyG index, triglyceride–glucose index; FIB-4, Fibrosis-4 score; eGFR, estimated glomerular filtration rate. Risk categories (scores 2–10) represent increasing levels of cumulative metabolic burden.

**Table 5:** Statistical comparison of TyG, FIB-4, and eGFR values across metabolic risk categories.

Variable	ANOVA *p*-Value	Kruskal–Wallis *p*-Value	Significance
TyG index	1.7 × 10^−20^	7.8 × 10^−21^	significant differences (progressively increases with risk)
FIB-4	4.8 × 10^−6^	6.1 × 10^−7^	significant (more advanced liver fibrosis at high risk)
eGFR	0.15	0.13	nonsignificant (decreasing trend with no clear differences)

ANOVA and Kruskal–Wallis tests were used to evaluate differences across metabolic risk categories. TyG index, triglyceride–glucose index; FIB-4, Fibrosis-4 score; eGFR, estimated glomerular filtration rate. Statistically significant *p*-values (<0.05) indicate a progressive increase in TyG and FIB-4 with rising metabolic risk, while eGFR showed a nonsignificant downward trend.

**Table 6:** Multivariate linear regression coefficients for predictors of FIB-4 score (Model A).

Variable	β (Unstandardized)	SE	β_st_d (Standardized)	*p*-Value	95% CI (Lower)	95% CI (Upper)
Constant	1.323	0.974	—	<0.001	−0.586	3.233
TyG index	−0.060	0.082	−0.034	<0.001	−0.221	0.101
Age	0.039	0.008	0.333	<0.001	0.024	0.054
Sex (2 = female)	−0.126	0.161	−0.049	<0.001	−0.442	0.190
BMI	−0.030	0.015	−0.106	<0.001	−0.059	−0.000
Systolic BP	0.001	0.003	0.017	<0.001	−0.005	0.007
HDL-C	−0.015	0.008	−0.109	<0.001	−0.031	0.002
eGFR	0.000	0.003	0.004	<0.001	−0.005	0.005
UACR	0.001	0.001	0.023	<0.001	−0.002	0.003

Dependent variable: FIB-4. Independent variables include TyG index, age, sex, BMI, systolic blood pressure, HDL-C, eGFR, and UACR. β, regression coefficient; SE, standard error; β_st_d, standardized beta; CI, confidence interval; TyG index, triglyceride–glucose index; BMI, body mass index; HDL-C, high-density lipoprotein cholesterol; eGFR, estimated glomerular filtration rate; UACR, urinary albumin-to-creatinine ratio. *p*-values < 0.05 were considered statistically significant.

**Table 8:** Multivariate linear regression coefficients for predictors of eGFR (Model B).

Variable	β (Unstandardized)	SE	β_st_d (Standardized)	*p*-Value	95% CI (Lower)	95% CI (Upper)
Constant	227.071	35.699	0.000	<0.001	157.102	297.040
TyG index	0.075	0.913	0.004	<0.001	−1.714	1.864
Age	−9.305	4.260	−0.296	<0.001	−17.654	−0.955
Sex (2 = female)	−0.973	0.137	−0.466	<0.001	−1.242	−0.704
BMI	7.559	2.741	0.163	<0.001	2.187	12.932
Systolic BP	−0.588	0.274	−0.117	<0.001	−1.124	−0.051
HDL-C	0.024	0.061	0.021	<0.001	−0.097	0.144
eGFR	−0.056	0.028	−0.121	<0.001	−0.111	−0.002
UACR	−0.033	0.153	−0.014	<0.001	−0.333	0.267
Variable	0.034	0.012	0.224	<0.001	0.010	0.058
Constant	0.034	0.042	0.082	<0.001	−0.048	0.116

Dependent variable: eGFR. Independent variables include FIB-4, TyG index, age, sex, BMI, systolic blood pressure, HDL-C, UACR, and related metabolic parameters. β, regression coefficient; SE, standard error; β_st_d, standardized beta; CI, confidence interval; TyG index, triglyceride–glucose index; BMI, body mass index; HDL-C, high-density lipoprotein cholesterol; eGFR, estimated glomerular filtration rate; UACR, urinary albumin-to-creatinine ratio; BP, blood pressure. *p*-values < 0.05 were considered statistically significant.

**Table 9:** Variance Inflation Factors (VIF) for predictors included in the multivariate regression model for eGFR (Model B).

Variable	VIF
FIB-4	1.20
TyG index	6.63
Age	1.44
Sex	1.16
BMI	1.18
Systolic BP	1.22
UACR	1.13
HDL-C	1.38
Triglycerides	3.74
Fasting Glucose	2.80

VIF, variance inflation factor; FIB-4, Fibrosis-4 score; TyG index, triglyceride–glucose index; BMI, body mass index; BP, blood pressure; UACR, urinary albumin-to-creatinine ratio; HDL-C, high-density lipoprotein cholesterol. Moderate multicollinearity was observed among metabolic variables, particularly TyG index (VIF = 6.63), triglycerides (VIF = 3.74), and fasting glucose (VIF = 2.80); however, all values remained below the critical threshold of 10, indicating model stability.

## Data Availability

The data presented in this study are available on request from the corresponding author. The data are not publicly available due to privacy or ethical restrictions.

## Supplementary Materials

The following supporting information can be downloaded at: https://www.mdpi.com/article/10.3390/metabo15110729/s1. Table S1. Comparison of TyG index, FIB-4 score, and eGFR across metabolic risk categories.

## Abbreviations

The following abbreviations are used in this manuscript:

ALT	Alanine aminotransferase
ANOVA	Analysis of variance
AST	Aspartate aminotransferase
BMI	Body mass index
BP	Blood pressure
CI	Confidence interval
CKD	Chronic kidney disease
CKD-EPI	Chronic Kidney Disease Epidemiology Collaboration
eGFR	Estimated glomerular filtration rate
FIB-4	Fibrosis-4 index
HDL-C	High-density lipoprotein cholesterol
HC3	Heteroscedasticity-consistent type 3
IQR	Interquartile range
MAFLD	Metabolic-associated fatty liver disease
SD	Standard deviation
SBP	Systolic blood pressure
TG	Triglycerides
TyG	Triglyceride–glucose index
UACR	Urinary albumin-to-creatinine ratio
VIF	Variance inflation factor
