# Supplementary material for: Integrative Assessment of TyG Index, FIB-4, and eGFR as Composite Predictors of Metabolic Risk Clusters in Adults
Source: Metabolites. 2025 Nov 7;15(11):729. doi: 10.3390/metabo15110729 (PMC12654196; doi:10.3390/metabo15110729)
Supplement: Supplementary file 1 [file metabolites-15-00729-s001.zip › metabolites-3963916-supplementary.pdf]

Table S1. Comparison of TyG index, FIB-4 score, and eGFR across metabolic risk categories.

Risk Category	n	TyG Index (Mean ± SD)	FIB-4 (Mean ± SD)	eGFR (Mean ± SD, mL/min/1.73 m²)
2	2	8.33 ± 0.11	0.89 ± 0.35	100.5 ± 2.1
3	13	8.32 ± 0.22	0.95 ± 0.33	100.6 ± 20.8
4	16	8.63 ± 0.44	1.23 ± 0.63	91.2 ± 33.6
5	44	8.80 ± 0.38	1.19 ± 0.50	83.8 ± 20.1
6	67	9.25 ± 0.64	1.14 ± 0.49	86.3 ± 21.6
7	81	9.53 ± 0.75	1.50 ± 1.67	84.0 ± 25.0
8	40	9.70 ± 0.60	1.58 ± 0.85	79.9 ± 21.4
9	16	9.76 ± 0.62	2.71 ± 2.47	76.2 ± 18.7
10	7	10.05 ± 0.46	3.37 ± 2.65	79.9 ± 26.0

ANOVA p-values: TyG = 1.7×10^{-20} (significant), FIB-4 = 4.8×10^{-6} (significant), eGFR = 0.15 (n.s.). Kruskal–Wallis p-values: TyG = 7.8×10^{-21}, FIB-4 = 6.1×10^{-7}, eGFR = 0.13. TyG and FIB-4 increase progressively with higher metabolic risk, whereas eGFR shows a mild, non-significant decline.
